# Efficacy of ultrasonics and Er,Cr:YSGG laser on root surface calculus removal: A comparative in vitro field emission scanning electron microscope study

**DOI:** 10.34172/japid.2024.008

**Published:** 2024-04-28

**Authors:** Afreen Jannath, Rajasekar Sundaram, Suganya Selvarangam, Krishnan Viswanathan, Srinivasan Sivapragasam

**Affiliations:** ^1^Department of Periodontology, Government Dental College and Hospital, Cuddalore District, Annamalai Nagar, Chidambaram, Tamil Nadu, India; ^2^Private Practice, Cuddalore, Tamil Nadu, India

**Keywords:** Dental calculus, Laser therapy, Root planing, Ultrasonics

## Abstract

**Background.:**

Scaling and root planing (SRP) is an inevitable primary step in non-surgical periodontal therapy. Debridement carried out with manual instruments and ultrasonics results in the removal of tooth structure. Current research revolves around laser as an efficient adjunct to SRP. This study evaluated and compared the effectiveness of root surface calculus removal between ultrasonics and Er,Cr:YSGG laser.

**Methods.:**

Twenty-eight single-rooted teeth extracted due to periodontal disease were selected for the study. The specimens were randomly assigned to two groups (n=14). Group I underwent ultrasonic instrumentation using a piezo ultrasonic scaler, and group II was subjected to laser instrumentation using Er,Cr:YSGG laser (Waterlase). The specimens were processed, fixed, viewed under a field emission scanning electron microscope and evaluated using the remaining calculus index (RCI) and loss of tooth substance index (LTSI).

**Results.:**

Ultrasonics-treated specimens revealed more remaining calculus (1.57±0.65) and lost tooth substance (1.71±0.61) compared to the Er,Cr:YSGG laser-treated specimens, with significantly lower RCI (0.71±0.61) and LTSI (1.00±0.56). There was a statistically significant difference (*P*<0.05) in the efficacy of root surface calculus removal between the two groups.

**Conclusion.:**

Compared to ultrasonics, Er,Cr:YSGG laser demonstrated superior results by causing precise removal of root surface calculus without significantly affecting tooth structure and aiding in new attachment.

## Introduction

 The primary goal of phase I periodontal therapy is to eliminate local factors and create a favorable root surface to facilitate new attachment of the lost periodontium.^1^ Scaling and root planing (SRP) is an inevitable primary step in non-surgical periodontal therapy.^2^ Debridement carried out with manual scalers and curettes provides tactile sensation but causes inadvertent removal of the root surface, with no access to the furcal area. Power-driven scalers, including ultrasonics, have facilitated the procedure with improved efficiency but cause vibration and noise.^1^

 Laser-assisted periodontics^3^ has been used for almost all periodontal therapies, from minor soft tissue surgeries to debridement of hard tissues like SRP, root biomodification, and bone harvesting. Because of the flexible and thin fibers, they can be easily delivered to deep periodontal pockets where conventional instruments are difficult to reach. Moreover, laser irradiation provides detoxification and bactericidal effects. Hence, laser application provides favorable conditions for new attachments.^4^

 Er,Cr:YSGG laser is an all-tissue laser that contains an active medium of YSGG crystals doped with Er and Cr. It is currently used in periodontal and peri-implant therapy.^5^ This laser utilizes fiber optics transmission and needs an additional air-water spray to be operated in free-running pulsed mode on dental tissues.^3^ Er,Cr:YSGG lasers emit photons at a 2.78-μm wavelength, similar to Er:YAG laser (2.94 μm).^5^ These photons are absorbed by water in the soft or hard tissues and cause micro-explosion of water, producing photoacoustic ablation on the tissue surface.^6,7^

 In the microscopic evaluation of the root planed surface, any remnant calculus and tooth substance loss acts as a niche for further periodontal disease progression and, therefore, the remaining calculus index (RCI) and loss of tooth substance index (LTSI), formulated by Meyer and Lie in 1977, can be used to evaluate and compare the effectiveness of instrumentation.^8^

 This in vitro study evaluated and compared the efficacy of root surface calculus removal and tooth substance loss between ultrasonics and Er,Cr:YSGG laser using field emission scanning electron microscopy (FESEM).

## Objectives

1. To evaluate and compare the efficiency of calculus removal between ultrasonics and Er,Cr:YSGG laser using RCI.^8^
2. To evaluate and compare the lost tooth substance between ultrasonics and Er,Cr:YSGG laser using LTSI.^8^

## Methods

###  Armamentarium 

 Single-rooted extracted teeth, 0.9% NaCl, distilled water, 4 ºC refrigerator, ultrasonics, Er,Cr:YSGG LASER, 2.5% glutaraldehyde dissolved in 0.1-M phosphate-buffered solution in a pH range of 7‒7.4, Airoter (high-speed rotary), ascending grades of alcohol: 30%, 50%, 70%, 90%, 100% concentrations, chromium sputter, field emission scanning electron microscope.

###  Sample size calculation

 Based on the article by Mishra and Prakash,^9^ the sample size was calculated at n = 14 per group based on G*Power Version 3.1.9.4 statistical software, maintaining an α error of 0.05 at 95% CI and a 95% power of the test (1-β error).

###  Sample collection 

 The present study was conducted in the Department of Periodontology, Government Dental College and Hospital, Cuddalore district, with the assistance of the Centralised Instrumentation and Service Laboratory, Annamalai University, from August to December 2022. Twenty-eight single-rooted teeth extracted due to periodontal disease were chosen for the study. Teeth extracted due to carious lesions, fractures, filling, or root canal therapy were excluded.Care was taken not to damage the tooth structure during the extraction. Following extraction, the teeth were washed in distilled water and kept in 0.9% NaCl solution until the start of the treatment.^9^

 Specimens were randomly assigned by flipping a coin to two groups (n = 14). A test area was cut on the proximal surface of the tooth by making two grooves 5 mm apart in the faciolingual direction below the cementoenamel junction. The sample collection, preparation, randomization, and instrumentation were carried out by a single trained operator (AJ).

###  Instrumentation of the test area

 Group I underwent ultrasonic instrumentation using a piezo ultrasonic scaler (Woodpecker UDS-P LED, Guilin Woodpecker Medical Instrument Co., Ltd. Information Industrial Park, Guilin National High-Tech Zone, Guilin, Guangxi,541004, China) tip 201. Scaling was performed using 30 strokes in an apicocoronal direction, using linear oscillations at a 30-kHz frequency.

 Group II underwent Er,Cr:YSGG laser irradiation (Waterlase Iplus, Biolase Inc, Foothill Ranch, CA, USA), carried out using Radial Firing Perio Tip 5, placed at a right angle to the long axis of the tooth. The Er,Cr:YSGG laser specifications were: wavelength = 2,780 nm, power = 3.50 W, and frequency = 75 Hz. The air-water spray ratio was 20:40. Three passes were made.

 After instrumentation, the treated root surfaces of the specimens in both groups were viewed under light and then examined with explorer #17/23 for the criteria of adequate treatment.

###  FESEM analysis and scoring

 The specimens were immersed in freshly prepared glutaraldehyde in 0.1-M phosphate-buffered solution at a pH of 7.4 for 24 hours and then rinsed in distilled water. The samples were dehydrated in increasing concentrations of ethyl alcohol (30% for 2 hours, 50% for 4 hours, 70% for 8 hours, 90% for 12 hours, and 100% for 24 hours), followed by 48 hours of air drying.

 The specimens were then fixed to FESEM stubs, sputter-coated with 2 nm of chromium in Quorum Q150T S PLUS, and then examined using FE-SEM Sigma – 300 from ZEISS. The standard photomicrographs of the test sites were obtained at × 100 and × 500 magnifications for each specimen. These photographs were then interpreted for RCI and LTSI by another examiner (SS) blinded from the study.

 The scoring system for RCI is presented in Table 1,^8^ and LTSI is presented in Table 2.^8^

**Table 1:** Scoring criteria for the remaining calculus index^8^

**Score**	**Description**
0	No calculus remaining on the root surface
1	Small patches of extraneous material, probably consisting of calculus
2	Definite patches of calculus confined to smaller areas
3	Considerable amounts of remaining calculus appearing as one or a few voluminous patches or as several smaller patches scattered on the treated surface

**Table 2:** Scoring criteria for Loss of Tooth Substance Index^8^

**Score**	**Description**
0	No detectable loss of tooth substance
1	Slight loss of tooth substance restricted to localized areas. Most of the cementum is intact
2	Definite loss of tooth substance on most of the treated surface, but without deep instrumental marks in the dentin. Cementum may be absent in some areas
3	Considerable loss of tooth substance with deep instrumental marks in the dentin. Most of the cementum is removed

###  Statistical analysis

 The efficacy of root surface calculus removal and lost tooth substance was analyzed using SPSS 21 (SPSS Inc., Chicago, IL, USA). Each group was considered an independent variable, and the non-parametric Mann-Whitney U test was employed for analysis.

## Results

###  Remaining calculus index

**Figure 1:**
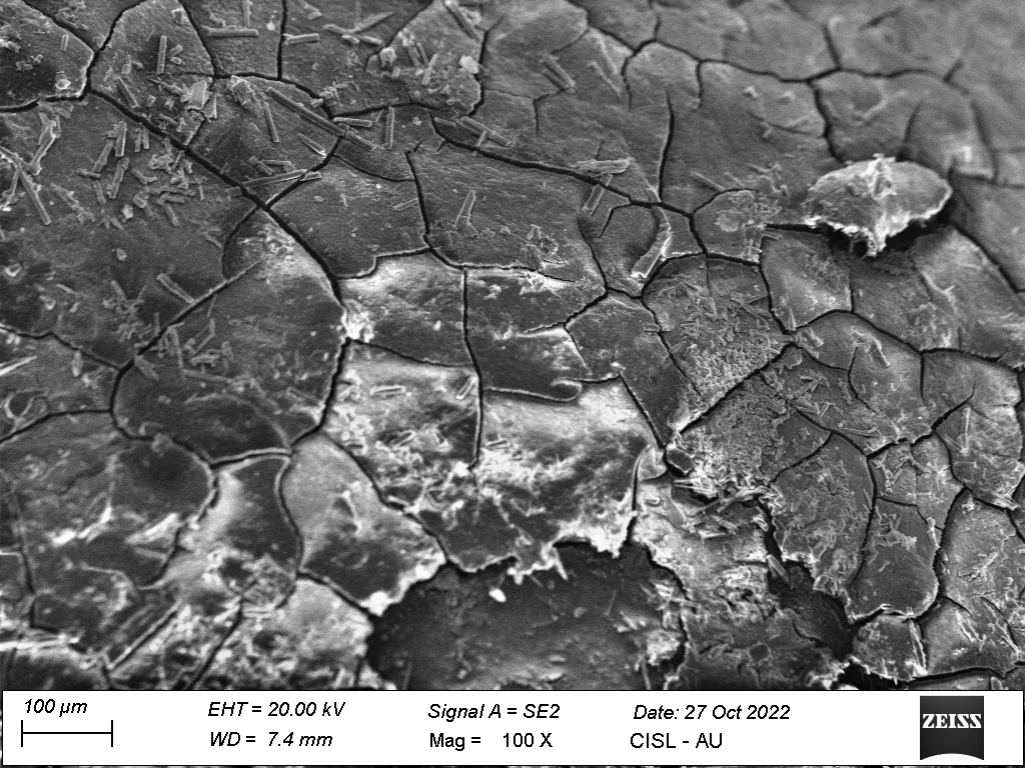
FESEM image illustrating definite patches of calculus in group I treated with ultrasonics (magnification: × 100)

*Group I:* Of 14 samples,seven samples showed small patches of extraneous material, probably calculus (score 1), six samples showed definite patches of calculus (score 2), and one sample showed considerable amounts of remaining calculus (score 3) (Figure 1).

**Figure 2:**
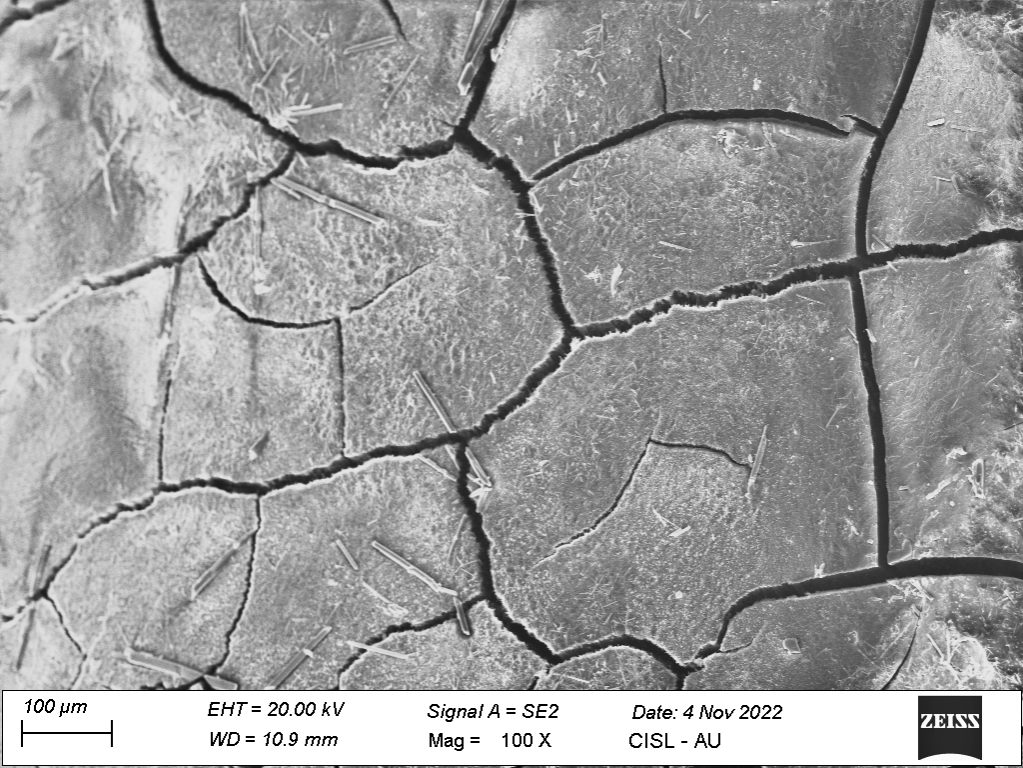
FESEM image illustrating very few specks of extraneous material, suggestive of calculus in group II treated with Er,Cr:YSGG laser (magnification: × 100)

*Group II:* Of 14 samples, five samples showed no calculus on the root surface (score 0), eight samples showed small patches of extraneous material, probably calculus (score 1), and one sample showed definite patches of calculus in small areas (score 2) (Figure 2).

**Figure 3:**
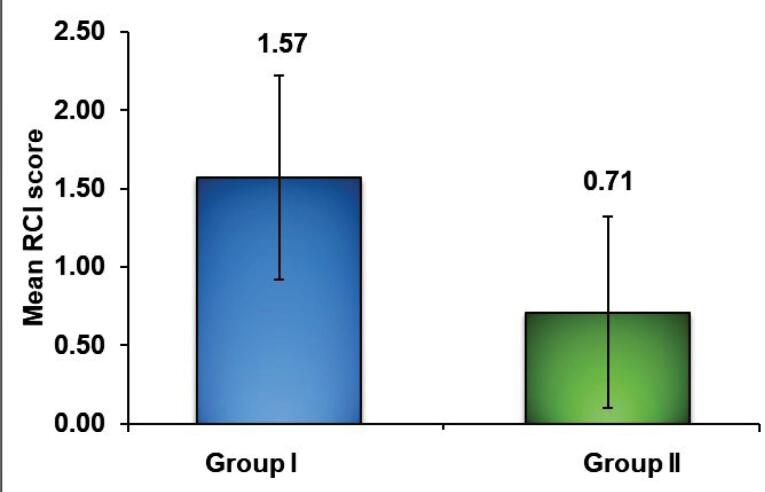
Graph depicting mean remaining calculus index scores in the study groups post FESEM analysis

 The mean ± standard deviation of RCI in group I was 1.57 ± 0.65, with 0.71 ± 0.61 in group II, yielding a statistically significant difference (*P* = 0.002) between the two groups (Table 3 and Figure 3).

**Table 3:** Intergroup comparison of remnant calculus using the remaining calculus index in post FESEM analysis

**Group**	**Mean±SD**	**95% confidence interval**	**U-value**	**Z-value**	***P*** ** value**
Group I (ultrasonic) (n = 14)	1.57 ± 0.65	1.20-1.94	38.000	-3.033	0.002*
Group II (Er,Cr:YSGG laser) (n = 14)	0.71 ± 0.61	0.36-1.07			

The scoring values of the remaining calculus index are presented as mean ± SD. Test applied: Mann-Whitney U test; significance level: * *P* ≤ 0.05 is considered statistically significant.

###  Loss of tooth substance index

**Figure 4:**
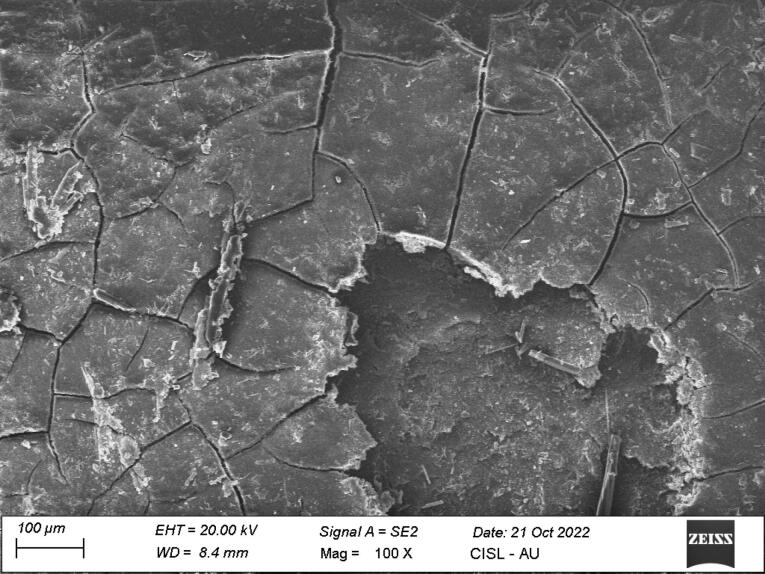
FESEM image illustrating definite loss of tooth substance with cementum absent in some areas in group I treated with ultrasonics (magnification: × 100)

*Group I:* Eight samples showed definite loss of tooth substance (score 2), five samples showed slight loss of tooth substance in localized areas (score 1), and one sample showed considerable loss of tooth substance with deep instrumental marks (score 3) (Figure 4).

**Figure 5:**
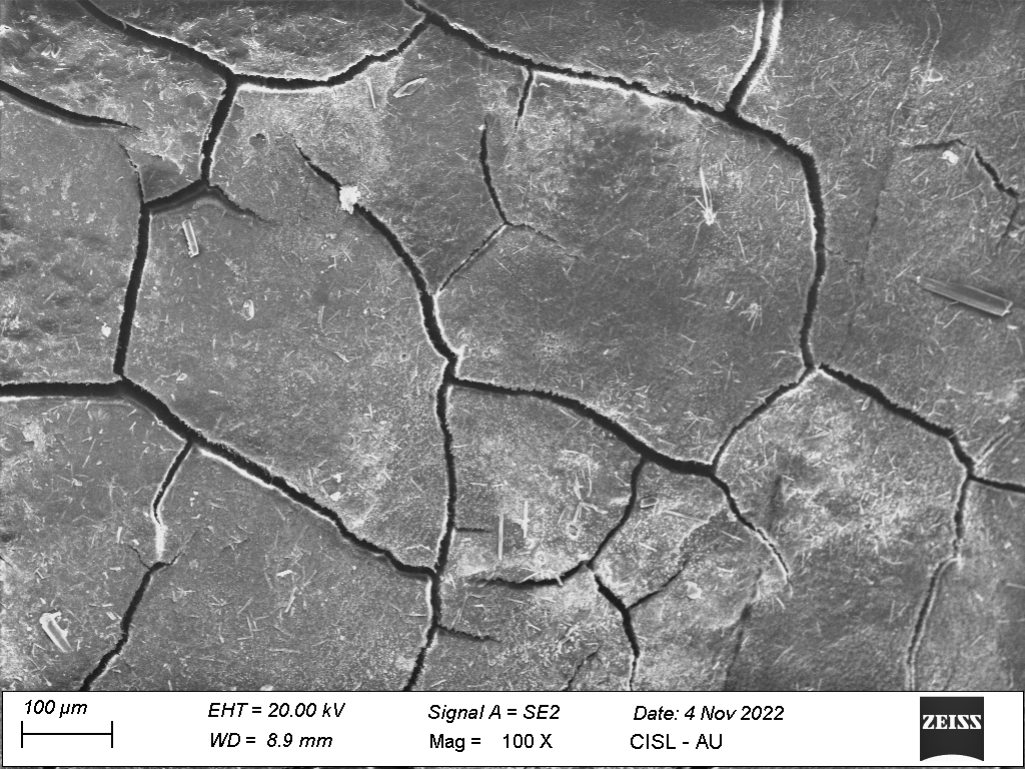
FESEM image illustrating the slight loss of tooth substance with most cementum intact in group II treated with Er,Cr:YSGG laser (magnification: × 100)

*Group II:* Two samples showed no detectable loss of tooth substance (score 0), ten samples showed slight loss of tooth substance in localized areas (score 1), and two samples showed definite loss of tooth substance (score 2) (Figure 5).

**Figure 6:**
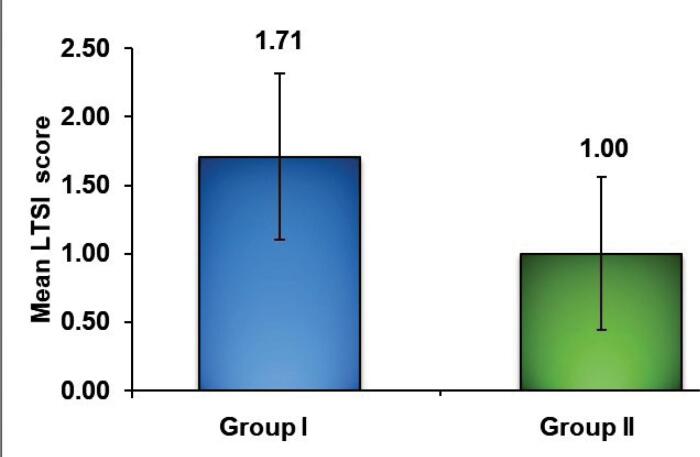
Graph depicting the mean loss of tooth substance index scores in the study groups post FESEM analysis

 The mean ± standard deviation of LTSI in group I was 1.71 ± 0.61, with 1.00 ± 0.56 in group II, revealing a statistically significant difference (*P* = 0.005) between the two groups (Table 4 and Figure 6).

**Table 4:** Intergroup comparison of lost tooth substance using the loss of tooth substance index in post FESEM analysis

**Group**	**Mean±SD**	**95% confidence interval**	**U-value**	**Z-value**	***P*** ** value**
Group I (ultrasonic) (n = 14)	1.71 ± 0.61	1.36‒2.07			
Group II (Er,Cr:YSGG laser) (n = 14)	1.00 ± 0.56	0.68‒1.32	43.000	-2.823	0.005*

The scoring values of the loss of tooth substance index are presented as mean ± SD. Test applied: Mann-Whitney U test; significance level: * *P* ≤ 0.05 is considered statistically significant

## Discussion

 Several modalities, including manual scalers, curettes, ultrasonics, lasers, etc., can be used for SRP. This is accomplished by removing local factors and providing an environment conducive for new attachment of periodontal tissues.^1^ Numerous studies have stated that using hand instruments with or without ultrasonics is equally efficient in eliminating local factors.^10-12^ Marda et al,^13^ in their in vitro study, compared manual, ultrasonic, and rotary instrumentation on root surfaces and reported that using ultrasonics yielded better results. Extensive research has well established the efficacy of Er:YAG lasers on calculus removal,^14-16^ but studies on Er,Cr:YSGG laser’s efficacy on calculus removal have reported contradictory results.^17-19^ The present study was designed to compare the effectiveness of Er, CR:YSGG laser with ultrasonics regarding RCI and LTSI, as given by Lie and Meyer^8^ in 1977.

 There was a statistically significant difference in RCI and LTSI between the two groups in the present study. The root surface treated with Er,Cr:YSGG laser exhibited better calculus removal with the least tooth substance loss, consistent with similar studies.^18,20^ Agoob Alfergany et al^18^ and Hakki et al,^20^ in their experiments with Er,Cr:YSGG laser, reported that the laser-treated surfaces exhibited more appropriate removal of residual debris with no thermal effect on the micromorphology on the root surface, which was similar to the results of the present study. Do Nascimento Tsurumaki et al,^21^ in their in vitro study, concluded that the ultrasonics and Er,Cr:YSGG lasers revealed no difference concerning the adhesion of blood components on the root surface.

 The Er,Cr:YSGG laser application at 1.0-W power promoted the highest level of attachment of blood components on root surfaces, allowing for optimal periodontal repair and regeneration. This was suggested by de Oliveira et al.^22^ They also stated that the Er,Cr:YSGG laser may be used safely for periodontal therapy.

 Amid et al^23^ and Aoki et al^16^ reported in their in vitro studies that the Er:YAG laser-irradiated samples exhibited more distortions and thermal microchanges on the root cementum, respectively. Etemadi et al^24^ compared root surface morphology after Er,Cr:YSGG and Er:YAG laser scaling and reported that in terms of calculus removal efficiency per power, both groups revealed no significant difference. Arora et al,^19^ in their in vitro study, concluded that the Er,Cr:YSGG lasers produced comparatively rougher surfaces that promoted plaque and calculus deposition and attachment of periodontal tissues, which was contradictory to the results of the present study.

 Several clinical studies have also reported that erbium lasers are more efficient for the attachment of periodontal tissues,^25–27^ which is consistent with the reports of the present study. Ertugrul et al^28^ reported that the Er,Cr:YSGG laser, as an adjunct in SRP, resulted in less Interleukin-1β and human β defensin-1 levels in generalized aggressive periodontitis and chronic periodontitis patients. Kelbauskiene et al,^29^ in a pilot study on the use of Er,Cr:YSGG laser in addition to SRP in early and moderate periodontitis patients, reported that the combined treatment using laser seemed to be more appropriate than SRP alone. Their one-year follow-up revealed significant improvements in all the periodontal clinical parameters in the group undergoing combined treatment.^27^

 Torkzaban et al,^30^ in a split-mouth randomized controlled trial, reported that the Er,Cr:YSGG laser-assisted periodontal flap surgery produced therapeutic results comparable to that of conventional treatment; thus, it was regarded as a reliable and secure treatment approach.

 The increased efficacy of Er,Cr:YSGG laser in the present study is attributed to the photoacoustic ablation activity of the laser, which interacts with the atomized water droplets and other internal components within calculus and produces hard tissue cuts, removing deposits without affecting tooth substance.The ablation effect is enhanced by water spray directed at the ablation site.

 Possible limitations of the study include only a single examiner who was blinded to the study (SS) and scored the specimens after FESEM analysis, which might have led to bias. It was an in vitro study, and the oral cavity could not be precisely simulated. Hence, the clinical correlations might not be certain. Variations in technique, operator, and instrument designs and handling may also contribute.^5,9^ Thus, future recommendations include conducting randomized controlled clinical trials on larger sample sizes with long-term follow-ups for further validation of the results.

## Conclusion

 Based on the findings of this study, Er,Cr:YSGG laser demonstrated superior results compared to ultrasonics by precise removal of root surface calculus without significantly affecting tooth substance loss and aiding in new attachment.

## Acknowledgments

 We would like to acknowledge and thank Laser India Academy for supporting our study with Waterlase technology.

## Competing Interests

 The authors declare no financial and non-financial competing interests with regard to the publication of their work during submission.

## Consent for Publication

 Not applicable.

## Data Availability Statement


The master chart has been submitted as Supplementary file 1.

## Ethical Approval

 The in vitro study was conducted on extracted human teeth. The study did not have any ethical registrations based on the recommendations of the Institutional Scientific Committee

## Funding

 This research received no specific grant from funding agencies in the public, commercial, or not-for-profit sectors.
