# Supplementary material for: Efficacy of ultrasonics and Er,Cr:YSGG laser on root surface calculus removal: A comparative in vitro field emission scanning electron microscope study
Source: J Adv Periodontol Implant Dent. 2024 Apr 28;16(1):9–14. doi: 10.34172/japid.2024.008 (PMC11252148; doi:10.34172/japid.2024.008)
Supplement: Supplementary file 1 — The master chart has been submitted as Supplementary file 1. [file japid-16-9-s001.pdf]

Jannath et al, *J Adv Periodontol Implant Dent,* 2024, 16(1), 9-S1
doi: 10.34172/japid.2024.008
https://japid.tbzmed.ac.ir

MASTER CHART

GROUP I- ULTRASONIC GROUP

SPECIMEN NO.	RCI SCORES	LTSI SCORES
1	2	2
2	2	3
3	1	2
4	1	1
5	3	2
6	2	1
7	2	2
8	1	1
9	1	2
10	2	1
11	2	1
12	1	2
13	1	2
14	1	2

GROUP II- Er, Cr:YSGG LASER GROUP

SPECIMEN NO.	RCI SCORES	LTSI SCORES
1	0	1
2	1	1
3	1	1
4	0	0
5	2	1
6	1	2
7	1	1
8	1	1
9	1	1
10	0	1
11	1	2
12	0	1
13	1	1
14	0	0
